# Lentivirus-mediated RNA interference targeting the *H19* gene inhibits cell proliferation and apoptosis in human choriocarcinoma cell line JAR

**DOI:** 10.1186/1471-2121-14-26

**Published:** 2013-05-27

**Authors:** Li-Li Yu, Kai Chang, Lin-Shan Lu, Dan Zhao, Jian Han, Ying-Ru Zheng, Yao-Hua Yan, Ping Yi, Jian-Xin Guo, Yuan-Guo Zhou, Ming Chen, Li Li

**Affiliations:** 1 Department of Obstetrics and Gynecology, Daping Hospital, The Third Military Medical University, Chongqing 400042, China; 2 Molecular Biology Center, Institute of Surgery Research, Daping Hospital, The Third Military Medical University, Chongqing 400042, China

**Keywords:** H19, JAR cells, Choriocarcinoma, HES-1, DUSP5, IGF2

## Abstract

**Background:**

*H19* is a paternally imprinted gene that has been shown to be highly expressed in the trophoblast tissue. Results from previous studies have initiated a debate as to whether noncoding RNA *H19* acts as a tumor suppressor or as a tumor promotor in trophoblast tissue. In the present study, we developed lentiviral vectors expressing *H19*-specific small interfering RNA (siRNA) to specifically block the expression of *H19* in the human choriocarcinoma cell line JAR. Using this approach, we investigated the impact of the *H19* gene on the proliferation, invasion and apoptosis of JAR cells. Moreover, we examined the effect of *H19* knockdown on the expression of insulin-like growth factor 2 (*IGF2*), hairy and enhancer of split homologue-1 (*HES-1*) and dual-specific phosphatase 5 (*DUSP5*) genes.

**Results:**

*H19* knockdown inhibited apoptosis and proliferation of JAR cells, but had no significant impact on cell invasion. In addition, *H19* knockdown resulted in significant upregulation of *HES-1* and *DUSP5* expression, but not *IGF2* expression in JAR cells.

**Conclusions:**

The finding that *H19* downregulation could simultaneously inhibit proliferation and apoptosis of JAR cells highlights a putative dual function for *H19* in choriocarcinoma and may explain the debate on whether *H19* acts as a tumor suppressor or a tumor promotor in trophoblast tissue. Furthermore, upregulation of *HES-1* and *DUSP5* may mediate *H19* downregulation-induced suppression of proliferation and apoptosis of JAR cells.

## Background

*H19* is a paternally imprinted gene encoding a noncoding RNA [1]. As one of the first imprinted genes discovered, *H19* was initially identified as a tumor suppressor because embryo-derived tumor cells overexpressing *H19* exhibited growth retardation, morphological changes and abrogated clonogenicity in soft agar, as well as suppressed tumorigenicity in nude mice [2]. Downregulation of *H19* gene expression was identified as an early event in the formation of several tumor types [3-8]. Recently it was demonstrated that mice lacking *H19* showed an overgrowth phenotype, while transgenic mice overexpressing *H19* showed postnatal growth reduction [9].

In contrast, overexpression or loss of imprinting (LOI) of *H19* has been observed in a wide variety of tumors, including bladder carcinoma [10,11], epithelial ovarian cancer [12], esophageal cancer [13], lung cancer [14], breast adenocarcinoma [15,16], endometrial cancer [17], and invasive cervical carcinoma [18]. *H19* knockdown significantly decreased the clonogenicity and anchorage-independent growth property of several breast and lung cancer cell lines [19]. A link between *H19* and several tumorigenesis-related genes, such as *c-Myc*[19], *thioredoxin*[20] and *E2F1*[21], has been well established. Thus, it remains controversial whether *H19* functions as a tumor promotor or a tumor suppressor, and it is possible that *H19* plays differential roles depending on tissue type and/or developmental stage [13].

*H19* is highly expressed in trophoblast tissue, predominantly but not exclusively from the maternal allele [22,23]. Complete hydatidiform moles (CHM) showed high-level expression of *H19* from the paternal allele, while choriocarcinomas developing from CHMs had reduced numbers of *H19*-positive cells [23]. *H19* downregulation was also observed in gestational trophoblastic tumors [24]. These observations appear to support the notion that *H19* functions as a tumor suppressor in trophoblast tissue. However, Ariel I *et al*. found that H19 expression was found to be abundant, in a decreasing order, in the intermediate trophoblast (villous and interstitial), the cytotrophoblast, and the syncytiotrophoblast. And prominent expression of H19 was found in placental site trophoblastic tumor and gestational choriocarcinoma [25]. After choriocarcinoma-derived JEG-3 cells were subcutaneously injected into nude mice, a five-fold increase in *H19* RNA level was detected in the resulting tumors, and cells highly expressing *H19* were more tumorigenic [26]. Therefore, the exact role of *H19* in the trophoblast is yet undetermined.

In previous studies, we demonstrated that DNA methyltransferase inhibitor 5-aza-2′-deoxycytidine could demethylate the promoter region of the *H19* gene, upregulate the expression of *H19* transcript, and reduce the proliferation, migration and invasion properties of choriocarcinoma-derived JEG-3 cells [27,28]. As the demethylating effects of 5-aza-2′-deoxycytidine are unspecific, other genes may be demethylated in tandem and complicate the biological effects. To further clarify our understanding of the function of the *H19* gene in choriocarcinoma, we developed lentiviral vectors expressing *H19*-specific small interfering RNA (siRNA) for use in human choriocarcinoma cells. Considering that the human choriocarcinoma cell line JAR is more easily infected by lentiviruses than the cell line JEG-3 (our unpublished observation), JAR cells were used in the present study. Using the siRNA technology, we investigated the impact of *H19* knockdown on the proliferation, invasion, and apoptosis of JAR cells. Furthermore, we examined the effects of *H19* knockdown on the expression of the insulin-like growth factor 2 (*IGF2*), hairy and enhancer of split homologue-1 (*HES-1*), and dual-specific phosphatase 5 (*DUSP5*) genes, which were chosen based on the previous evidence that *H19* is a negative regulator of *IGF2*[29] and our unpublished observation that the expression of *HES-1* and *DUSP5* genes was altered in JEG-3 cells transfected with a eukaryotic expression vector carrying the full-length *H19* cDNA.

## Methods

### Cell culture

Human choriocarcinoma cell line JAR was purchased from American Type Culture Collection (USA). After thawing, cells were allowed to grow in Dulbecco’s modified Eagle’s medium/Ham’s nutrient mixture F12 (DMEM/F12; Gibco-BRL, USA) medium supplemented with 10% fetal bovine serum (Gibco-BRL, USA) at 37°C in a humidified atmosphere containing 5% CO_2_. After three passages, cells were used for viral infection.

### Construction and screening of lentiviral vectors harboring H19-specific siRNA

The following siRNA target sequences in the human *H19* gene (GenBank accession No. NR_002196) were selected: TARGET1, GCCTTCAAGCATTCCATTA; TARGET2, GGAGAGTTAGCAAAGGTGA; TARGET3, CGTGACAAGCAGGACATGA; and TARGET4, GAGGAACCAGACCTCATCA. Four pairs of complementary oligonucleotides were then designed (Additional file 1: Table S1). The stem-loop oligonucleotides were synthesized and cloned into a lentivirus-based vector carrying the green fluorescent protein (GFP) gene (pGCSIL-GFP, Genechem, Shanghai, China). A universal sequence (PSC-NC: TTCTCCGAACGTGTCACGT) was used as a negative control for RNA interference. Lentiviral particles were prepared as previously described [30].

The four siRNA-carrying lentiviral vector constructs were used to infect JAR cells at a multiplicity of infection (MOI) of 20 (low MOI) and 40 (high MOI). Three days after infection, GFP expression was detected to calculate the infection efficiency. Five days after infection, cells were harvested. Real-time reverse transcription-polymerase chain reaction (RT-PCR) was performed to determine *H19* knockdown efficiency and screen for the siRNA with the highest knockdown efficiency which was then used for subsequent experiments.

### RNA isolation, reverse transcription and quantitative PCR

Total RNA isolation using Trizol Reagent (Invitrogen, USA) and reverse transcription using Moloney murine leukemia virus (M-MLV) reverse transcriptase (Promega, USA) were carried out according to the manufacturer’s instructions. Quantitative PCR was performed in a final volume of 20 *μ*l containing 1 μl cDNA, 0.5 μl primers (2.5 μM) and 10 μl SYBR premix exTaq (TaKaRa, Dalian, China). The cycling conditions were: 95°C for 15 min; 40 cycles of 95°C for 5 s and 60°C for 30 s; and one cycle of 95°C for 60 s, 55°C for 60 s and 95°C for 60 s. The sequences of primers used in real-time RT-PCR were as follows: forward 5′-TCCCAGAACCCACAACATGAA-3′ and reverse 5′-TTCACCTTCCAGAGCCGATTC-3′ for *H19* (150 bp); forward 5′-TCCTCACCTCGCTACTCG-3′ and reverse 5′-ACATCCACGCAACACTCAG-3′ for *DUSP5* (106 bp); forward 5′-GAGAGGCGGCTAAGGTGTTTG-3′ and reverse 5′-CTGGTGTAGACGGGGATGAC-3′ for *HES-1* (121 bp); forward 5′-CCTCCAGTTCGTCTGTGGG-3′ and reverse 5′-CACGTCCCTCTCGGACTTG-3′ for IGF2 (163 bp); and forward 5′-GGCGGCACCACCATGTACCCT-3′ and reverse 5′-AGGGGCCGGACTCGTCATACT -3′ for beta-actin (202 bp). Data analysis was performed using the 2^-ΔΔCt^ method. The experiment was independently repeated three times.

### Cell cycle analysis

Cells were seeded onto six-well plates and cultured. Upon reaching 80% confluence, cells were released by digestion with trypsin and harvested. After centrifugation, the cell pellet was washed twice with pre-cooled phosphate buffered saline (PBS) and fixed with pre-cooled 70% ethanol. Then, cells were resuspended in PBS, filtered through a 400-mesh sieve, and stained with propidium iodide (PI, 50 μg/mL, 100 μg/mL RNase in PBS) at 37°C for 30 min. The cell cycle was then determined by flow cytometry as previously described [31].

### Apoptosis assay

Apoptosis was detected using the ApoScreen Annexin V Apoptosis Kit (SouthernBiotech, USA) according to the manufacturer’s instructions. In addition, one more group of cells was incubated with 30 μg/ml diaminedichloroplatinum (DDP) for 16 hours and subjected to apoptosis assay. The experiment was independently repeated three times.

### Methyl thiazolyl tetrazolium (MTT) assay

Cells were seeded in sextuplicate for each condition in 96-well plates at a density of 2×10^4^/mL in 100 μl per well. After 24 hours of culture, medium was replaced. Cells were then further cultured in this manner for 1–6 days. Four hours before the termination of culture, MTT (5 mg/mL; Sigma, USA) was added at a volume of 10 μL per well. Afterwards, the entire supernatant was discarded and dimethyl sulfoxide (DMSO) was added at a volume of 100 μL per well and incubated in an air bath shaker at 37°C for 10 min. The absorbance at 570 nm of each well was determined using the 1420 Multilabel Counter (PerkinElmer, USA). The experiment was independently repeated three times.

### Cell invasion assay

The cell invasion assay was performed using a commercial kit (Chemicon, Canada), according to the manufacturer’s instructions. The CHEMICON Cell Invasion Assay is performed in an Invasion Chamber, a 24-well tissue culture plate with 12 cell culture inserts. The inserts contain an 8 μm pore size polycarbonate membrane, over which a thin layer of ECMatrixTM is dried. The ECMatrixTM layer serves as an *in vitro* reconstituted basement membrane and occludes the membrane pores, blocking non-invasive cells from migrating through. Invasive cells, on the other hand, migrate through the ECM layer and cling to the bottom of the polycarbonate membrane. Before JAR cells were plated at a density of 1 × 10^4^ cells in each insert, 300 μL of warm serum free media were added to the interior of the inserts to rehydrate the ECM layer for 1–2 hours at room temperature. Cells were incubated at 37°C in 5% CO_2_ for 72 h. A cotton-tipped swab was used to gently remove non-invading cells as well as the ECMatrix gel from the interior of the inserts. Five hundred microliters of staining solution were added to the unoccupied wells of the plate. Invasive cells on lower surface of the membrane were stained by dipping inserts in the staining solution for 20 minutes. Inserts were dipped in a beaker of water several times to rinse and air dried. Stained cells were dissolved in 10% acetic acid and OD at 560 nm was detected by colorimetric reading. Each assay was performed in triplicate and repeated three times.

### Statistical analysis

Statistical analysis was performed using SPSS 13.0 software package (SPSS Inc, Chicago, IL, USA). Numerical data are expressed as mean ± standard deviation. Multiple means were compared using analysis of variance and the Tukey post hoc test, while two means were compared using Student’s *t*-test. A *P* value of less than 0.05 was considered statistically significant.

## Results

### Selection of the lentiviral vector harboring siRNA with the highest knockdown efficiency

**Figure 1:**
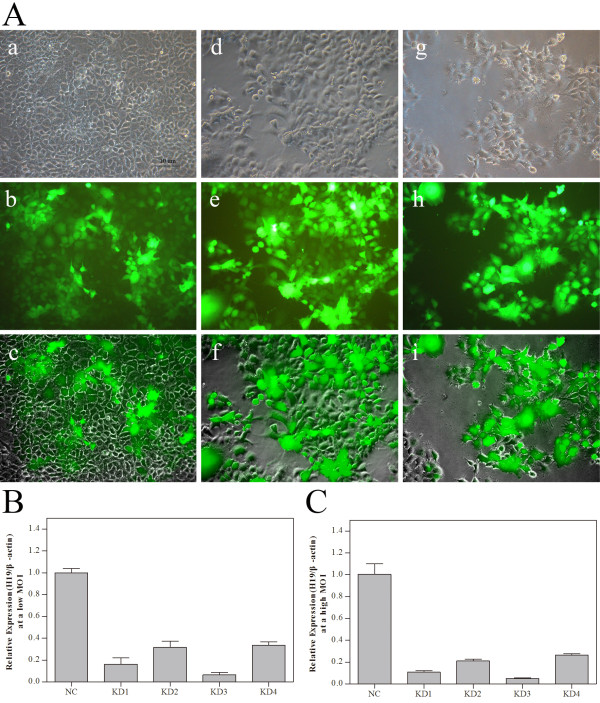
**Selection of the lentiviral vector harboring siRNA with the highest knockdown efficiency. A**, Fluorescence microscopy examination of the infection efficiencies of different lentiviral vectors in JAR cells (magnification 200 ×). a. JAR cells infected with lentiviral particles harboring a non-targeting control siRNA (NC group) in the light microscope; b. JAR cells of NC group in the fluorescence microscope; c. Fusion image of the top two images; d. JAR cells infected with KD3-harboring lentiviral particles (KD group)at a low MOI in the light microscope; e. JAR cells of KD group at a low MOI in the fluorescence microscope; f. Fusion image of the top two images; g. JAR cells infected with KD3-harboring lentiviral particles at a high MOI in the light microscope; h. JAR cells of KD group at a high MOI in the fluorescence microscope. i. Fusion image of the top two images. The fluorescence expression in cells infected with KD1, KD2 and KD4 was similar to that in cells infected with KD3. The infection efficiencies of these lentiviral vectors were about to be above 80%. **B**, Relative levels of *H19* in JAR cells infected with different groups of lentiviral particles at a low MOI. **C**, Relative levels of *H19* in JAR cells infected with different groups of lentiviral particles at a high MOI. Compared to the NC group, *H19* expression was significantly downregulated in JAR cells infected with different groups of lentiviral particles, at either a low or high MOI. Beta-actin was used to normalize the PCR data. The highest knockdown efficiency was achieved using KD3-harboring lentiviral particles.

The four lentiviral constructs harboring different siRNAs (KD1, KD2, KD3 and KD4) were used to infect JAR cells. In parallel, a negative control (NC) was run. The infection efficiencies of these lentiviral vectors were all above 80% as revealed by fluorescence microscopy (Figure 1A). Real-time RT-PCR assay showed that all four constructs, whether they were used at a high or low MOI, could significantly downregulate *H19* gene expression in JAR cells (Figure 1B, C). The highest knockdown efficiency was achieved using KD3 (low MOI: 93% relative to the NC group; high MOI: 95%), which was subsequently designated as *H19*-RNAi-LV-3.

### H19 Knockdown inhibited the proliferation of JAR cells

**Figure 2:**
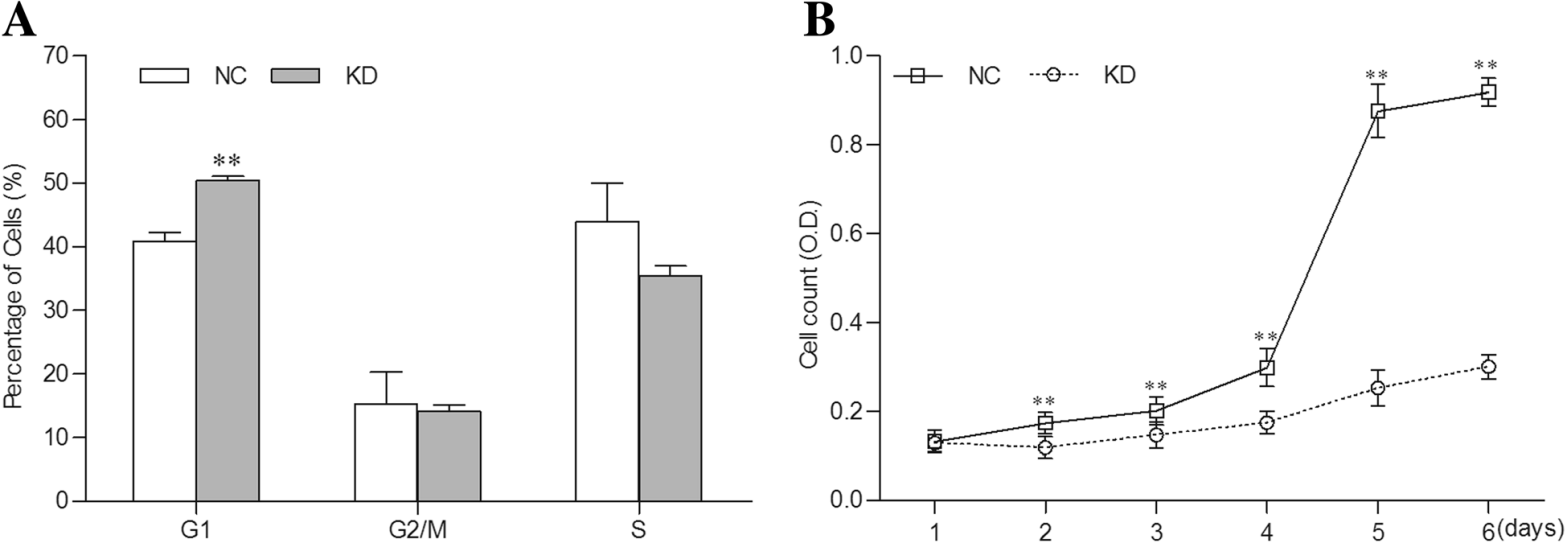
**Effect of *H19 *knockdown on the growth of JAR cells. A**, Comparison of the percentages of cells in G1, G2/M and S phase between NC and KD groups. The percentage of cells in G1 phase was significantly higher in the KD group than in the NC group, while the percentages of cells in G2/M phase and S phase respectively showed no significant changes between the two groups. **B**, Growth curves of cells in each group. MTT analysis showed that *H19* knockdown significantly inhibited cell proliferation (*n* = 18). ** denotes *P* < 0.01.

The percentage of cells in G1 phase was significantly higher in the *H19* knockdown group than in the NC group (*P* < 0.01), while the percentages of cells in G2/M phase and S phase respectively showed no significant changes between the two groups (Figure 2A). The MTT assay showed that cell proliferation was significantly inhibited in the *H19* knockdown group as compared to that in the NC group (*P* < 0.01) (Figure 2B).

### H19 Knockdown inhibited apoptosis of JAR cells

**Figure 3:**
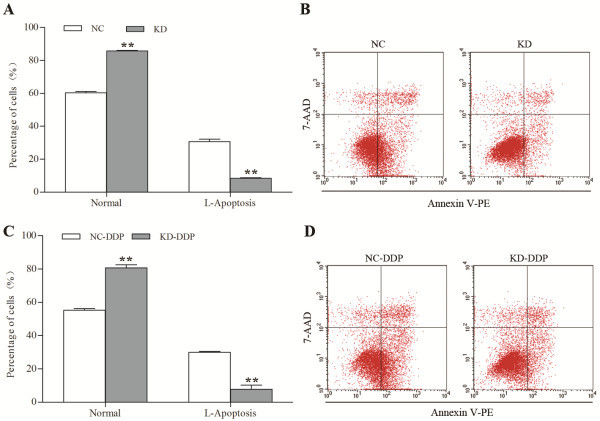
**Effect of *H19 *knockdown on the apoptosis of JAR cells. A**. Comparison of the percentages of normal cells and early apoptotic cells between the NC group and the KD group. *H19* knockdown significantly increased the percentage of normal cells but decreased the percentage of early apoptotic cells when compared with the NC group; **B**. Flow cytometric analysis of JAR cells in the NC group and the KD group; **C**. Comparison of the percentages of normal cells and early apoptotic cells between the two groups with DDP treatment. *H19* knockdown also significantly increased the percentage of normal cells but decreased the percentage of early apoptotic cells when compared with the NC-DDP group; **D**. Flow cytometric analysis of JAR cells in the NC-DDP group and the KD-DDP group. NC-DDP is the negative control vector-harboring cells incubated with DDP; KD-DDP, H19-RNAi-LV-3-infected cells incubated with DDP. ** denotes *P* < 0.01.

As shown in Figure 3, *H19* knockdown significantly increased the percentage of normal cells but decreased the percentage of early apoptotic cells when compared to the negative control group (85.70% ± 0.41% vs*.* 60.4% ± 0.70% and 8.46% ± 0.37% vs*.* 30.74% ± 1.45%, respectively; *Ps* < 0.01 for both). After incubation with diaminedichloroplatinum (an apoptosis inducer) for 16 hours, the percentage of normal cells was still significantly higher in the *H19* knockdown group than in the NC group (80.70% ± 1.88% *vs.* 55.20% ± 0.98%, *P* < 0.01), while the percentage of early apoptotic cells was still significantly lower in the *H19* knockdown group than in the NC group (7.80% ± 2.39% *vs.* 30.00% ± 0.46%, *P* < 0.01).

### H19 Knockdown does not alter the invasion of JAR cells

To determine if *H19* knockdown was able to affect the invasion properties of JAR cells, the cell invasion assay was performed. The invasion rate of cells in the *H19* knockdown group and the NC group were 68.85% ± 3.04% and 79.50% ± 5.52%, respectively. *H19* knockdown had no significant impact on the invasion rate of JAR cells. The result was shown in Additional file 1: Figure S1.

### H19 Knockdown upregulated HES-1 and DUSP5 expression but not IGF2 expression

**Figure 4:**
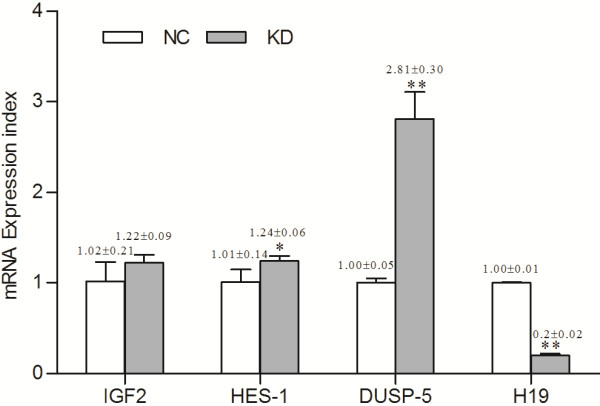
**Effect of *H19 *knockdown on the expression of *IGF2*, *HES-1 *and *DUSP5 *mRNAs in JAR cells.***H19* knockdown significantly upregulated the expression of *HES-1* and *DUSP5* mRNAs, but had no significant impact on the expression of *IGF2* mRNA. KD, *H19* knockdown group. * denotes *P* < 0.05 and ** denotes *P* < 0.01.

To gain insight into how *H19* knockdown alters the phenotype of human choriocarcinoma cells, we examined the expression of *IGF2*, *HES-1* and *DUSP5* mRNAs by real-time RT-PCR. The results showed that, compared with the negative control group, *H19* knockdown significantly upregulated the expression of *HES-1* and *DUSP5* mRNAs (1.24 ± 0.06 *vs.* 1.01 ± 0.14 and 2.81 ± 0.30 *vs.* 1.00 ± 0.05; *P* < 0.05 and 0.01, respectively), but had no significant impact on the expression of *IGF2* mRNA (1.22 ± 0.09 *vs.* 1.02 ± 0.21; *P* > 0.05, Figure 4). The Ct values were shown in Additional file 1: Table S2.

## Discussion

Using a lentiviral vector harboring *H19*-specific siRNA, the expression of *H19* in JAR cells was specifically downregulated. We showed that the invasion of JAR cells was not affected in response to *H19* downregulation, which is inconsistent with our previous studies indicating that 5-aza-2′-deoxycytidine-mediated *H19* upregulation could reduce the invasion capacity of JEG-3 cells [27,28]. This may be due to unspecific demethylating effects of 5-aza-2′-deoxycytidine or differences among the cell types studied. Furthermore, we also demonstrated that *H19* knockdown inhibited the apoptosis and proliferation properties of JAR cells. The results of the present study indicate that *H19* exerts both tumor-promoting and tumor-suppressing effects in choriocarcinoma cells, at least in part, by regulating cell proliferation and apoptosis.

It has been hypothesized for many years that a potential tumor suppressor gene is present at chromosome 11p15.5 where *H19* is located, because a loss of heterozygosity at this locus is observed in certain embryonal tumors [2]. Subsequent experiments implicated *H19* may function as a tumor suppressor [2,8]. However, other studies provided conflicting evidence that supported the idea that *H19* RNA harbors oncogenic properties [10-20]. Similar observations have been made in trophoblast tissue; some studies revealed that *H19* has tumor-suppressing activity [23,24], while others suggested that it has tumor-promoting activity [26]. Therefore, *H19* may play differential roles under different physiologic conditions. In the present study, we demonstrated that *H19* knockdown could simultaneously inhibit the proliferation and apoptosis of JAR cells, suggesting that *H19* expression may have both proliferation-inducing and apoptosis-inducing activity in choriocarcinoma-derived cells. Interestingly, a key event in tumorigenesis is the loss of equilibrium between cell proliferation and cell death [32]. Thus, our observation that *H19* can simultaneously induce proliferation and apoptosis highlights a putative dual role of *H19* in choriocarcinoma cell lines and may explain the debate over whether *H19* is a tumor suppressor or a tumor promotor in trophoblast tissue. Shoshani O *et al*. discovered that direct knockdown of H19 expression in diploid tumorigenic mouse mesenchymal stromal cells(MSCs) resulted in acquisition of polyploid cell traits. And artificial tetraploidization of diploid MSCs reduces tumorigenic potential in conjunction with suppression of H19 expression. This establishs a link between H19 expression and cell ploidy. Poluploidization might either have a cancer promoting or a cancer-preventing effect as is the case in aneuploidy [33]. Then, further experiments should determine whether the inhibition of proliferation and apoptosis by H19 knockdown is associated with the polyploidy of trophoblast cells. However, since the human choriocarcinoma cell line JAR may already have a different *H19* gene regulatory pattern from normal trophoblasts, the extrapolation of results from JAR cells to the normal trophoblast must be cautious. Further studies in normal trophoblast cells should be done to address this issue.

Like *H19*, *IGF2* is an imprinted gene and the two are closely linked. However, while *H19* is expressed from the maternal allele, *IGF2* is transcribed from the paternal allele in most tissues [34]. A growing body of evidence has indicated that *H19* RNA is a negative regulator of *IGF2*. Gabory *et al.*[9] demonstrated that *H19* transgenic or knockout mice developed abnormal phenotypes due to alterations in *IGF2* mRNA levels. Esquiliano *et al.*[35] found that targeted disruption of the *H19* gene in mice induced the placental phenotype via upregulation of *IGF2* expression. In the present study, we have demonstrated that *H19* knockdown had no significant impact on the expression of *IGF2* mRNA. Nevertheless, this result is not too surprising because numerous studies have not been able to provide a consistent relationship between the expression levels and imprinting status of *H19* and *IGF2* in all tissues. Adam *et al.*[36] discovered that *H19* showed biallelic expression in extravillous cytotrophoblasts, while *IGF2* showed monoallelic expression. Lustig-Yariv *et al.*[37] showed that *H19* and *IGF-2* RNA levels differed greatly among different JAR or JEG-3 clones. Moreover, we also found that both *H19* and *IGF-2* RNAs were highly expressed in normal term placenta and JEG-3 cells, and *H19* overexpression did not alter *IGF2* expression in JEG-3 cells (unpublished data). Therefore, these data suggest that *IGF2* may not be involved in *H19* downregulation-mediated inhibition of proliferation and apoptosis of JAR cells.

Our results did show that *H19* knockdown significantly upregulated expression of the *HES-1* gene in JAR cells. *HES-1* gene is an important effector of the Notch pathway that has been implicated in regulating the proliferation, differentiation and apoptosis in multiple tumor cell types. Kunnimalaiyaan *et al.*[38] found that increased *HES-1* expression suppressed the proliferation of carcinoid tumor cells. Nefedova *et al.*[39] demonstrated that overexpression of *HES-1* abrogated gamma-secretase inhibitor-induced apoptosis in multiple myeloma cells. Importantly, we also found that *H19* overexpression could downregulate the expression of *HES-1* in JEG-3 cells (about 2.27 fold) (unpublished data). Thus, we speculated that *HES-1* may mediate *H19* downregulation-induced suppression of proliferation and apoptosis of JAR cells. Although it remains unclear how *H19* knockdown upregulates *HES-1* expression in JAR cells, the recent finding that *H19* can negatively regulate an imprinted gene network may provide some clues to the mechanisms of regulation of *HES-1* expression by *H19*[9].

Furthermore, we demonstrated that *H19* knockdown could also significantly upregulate the expression of *DUSP5* gene in JAR cells. The *DUSP5* gene encodes a mitogen-activated protein kinase phosphatase (MKP). It has been shown that extracellular signal regulated kinase 2 (ERK2) is a specific substrate of *DUSP5*[40]. As ERK2 plays an important role in regulating cell proliferation and apoptosis, it is reasonable to surmise that *DUSP5* may also be involved in such processes. Importantly, we also found that *H19* overexpression could downregulate the expression of *DUSP5* in JEG-3 cells (about 2.63 fold) (unpublished data). Therefore, *DUSP*5 may also be involved in *H19* downregulation-induced suppression of proliferation and apoptosis of JAR cells.

## Conclusions

The present study provides evidence that *H19* knockdown does not alter invasion but inhibits proliferation and apoptosis of JAR cells. The observation that *H19* downregulation simultaneously suppresses proliferation and apoptosis of JAR cells suggests a putative dual functionality of *H19* in choriocarcinoma cell lines and may explain the debate over whether *H19* is a tumor suppressor or a tumor promotor in trophoblast tissue. Moreover, we also found that *H19* knockdown upregulates the expression of the *HES-1* and *DUSP5* genes but not of the *IGF-2* gene, suggesting that *HES-1* and *DUSP5* may mediate *H19* downregulation-induced suppression of proliferation and apoptosis of JAR cells.

## Abbreviations

CHM: Complete hydatidiform moles; DDP: Diamminedichloroplatinum; DMSO: Dimethyl sulfoxide; DUSP5: Dual-specific phosphatase 5; ERK2: Extracellular signal regulated kinase 2; GFP: Green fluorescent protein; HES-1: Hairy and enhancer of split homologue-1; IGF2: Insulin-like growth factor 2; LOI: Loss of imprinting; MKP: Mitogen-activated protein kinase phosphatase; M-MLV: Moloney murine leukemia virus; MOI: Multiplicity of infection; MTT: Methyl thiazolyl tetrazolium; NC: Negative control; PBS: Phosphate buffered saline; PI: Propidium iodide; RT-PCR: Reverse transcription-polymerase chain reaction; siRNA: Small interfering RNA

## Competing interests

The authors declare that they have no competing interests.

## Authors’ contributions

LL and MC contributed to the overall conceptual design and also analyzed the data, contributed to the interpretation of findings and wrote the first draft of the manuscript. LY and KC was the principal investigator and contributed with the idea, as well as to the interpretation of the results and the writing of the manuscript.CN, JS, FM, LM, AN, JK, GM, JO, LG, CR and BB were involved in the experiment of the study and revision of the manuscript. All authors have read and approved the final manuscript.

## Supplementary Material

Additional file 1: Table S1 The structure of siRNAs in lentiviral vectors. **Table S2** The Ct values from quantitative PCR reactions. **Figure S1**: Effect of *H19* knockdown on the invasion of JAR cells. The invasion rate of cells in the *H19* knockdown group and the NC group were 68.85% ± 3.04% and 79.50% ± 5.52%, respectively. *H19* knockdown had no significant impact on the invasion rate of JAR cells. Click here for file
