# Supplementary material for: Lentivirus-mediated RNA interference targeting the H19 gene inhibits cell proliferation and apoptosis in human choriocarcinoma cell line JAR
Source: BMC Cell Biol. 2013 May 27;14:26. doi: 10.1186/1471-2121-14-26 (PMC3679798; doi:10.1186/1471-2121-14-26)
Supplement: Additional file 1: Table S1 — The structure of siRNAs in lentiviral vectors. Table S2 The Ct values from quantitative PCR reactions. Figure S1: Effect of H19 knockdown on the invasion of JAR cells. The invasion rate of cells in the H19 knockdown group and the NC group were 68.85% ± 3.04% and 79.50% ± 5.52%, respectively. H19 knockdown had no significant impact on the invasion rate of JAR cells. [file 1471-2121-14-26-S1.doc]

**Supplementary material**

Table 1S The structure of siRNAs in lentiviral vectors

| NO. | 5’ | STEM | Loop | STEM | 3’ |
| --- | --- | --- | --- | --- | --- |
| TARGET1-1 | Ccgg | caGCCTTCAAGCATTCCATTA | TTCAAGAGA | TAATGGAATGCTTGAAGGCtg | TTTTTg |
| TARGET1-2 | aattcaaaaa | caGCCTTCAAGCATTCCATTA | TCTCTTGAA | TAATGGAATGCTTGAAGGCtg |  |
| TARGET2-1 | Ccgg | caGGAGAGTTAGCAAAGGTGA | TTCAAGAGA | TCACCTTTGCTAACTCTCCtg | TTTTTg |
| TARGET2-2 | aattcaaaaa | caGGAGAGTTAGCAAAGGTGA | TCTCTTGAA | TCACCTTTGCTAACTCTCCtg |  |
| TARGET3-1 | Ccgg | gaCGTGACAAGCAGGACATGA | TTCAAGAGA | TCATGTCCTGCTTGTCACGtc | TTTTTg |
| TARGET3-2 | aattcaaaaa | gaCGTGACAAGCAGGACATGA | TCTCTTGAA | TCATGTCCTGCTTGTCACGtc |  |
| TARGET4-1 | Ccgg | taGAGGAACCAGACCTCATCA | TTCAAGAGA | TGATGAGGTCTGGTTCCTCta | TTTTTg |
| TARGET4-2 | aattcaaaaa | taGAGGAACCAGACCTCATCA | TCTCTTGAA | TGATGAGGTCTGGTTCCTCta |  |

Table 2S The Ct values from quantitative PCR reactions

|  | | Actin Ct | IGF2 | | | |  | HES-1 | | | |  | DUSP5 | | | |
| --- | --- | --- | --- | --- | --- | --- | --- | --- | --- | --- | --- | --- | --- | --- | --- | --- |
|  |  |  | Ct | ΔCt | -ΔΔCt | 2^-ΔΔCt^ |  | Ct | ΔCt | -ΔΔCt | 2^-ΔΔCt^ |  | Ct | ΔCt | -ΔΔCt | 2^-ΔΔCt^ |
| 1 | **NC** | 13.65 | 30.83 | 17.18 | -0.297 | 0.814 |  | 25.25 | 11.6 | -0.023 | 0.984 |  | 23.1 | 9.45 | 0.050 | 1.035 |
|  | **NC** | 13.65 | 30.58 | 16.93 | -0.047 | 0.968 |  | 25.23 | 11.58 | -0.003 | 0.998 |  | 23.17 | 9.52 | -0.020 | 0.986 |
| 1 | **KD** | 13.1 | 29.65 | 16.55 | 0.333 | 1.260 |  | 24.44 | 11.34 | 0.237 | 1.179 |  | 20.98 | 7.88 | 1.620 | 3.074 |
|  | **KD** | 13.13 | 29.59 | 16.46 | 0.423 | 1.341 |  | 24.33 | 11.2 | 0.377 | 1.299 |  | 21.01 | 7.88 | 1.620 | 3.074 |
| 2 | **NC** | 14.18 | 30.94 | 16.76 | 0.363 | 1.286 |  | 25.2 | 11.02 | 0.340 | 1.266 |  | 23.11 | 8.93 | 0.103 | 1.074 |
|  | **NC** | 13.83 | 31.17 | 17.34 | -0.217 | 0.860 |  | 25.44 | 11.61 | -0.250 | 0.841 |  | 22.86 | 9.03 | 0.003 | 1.002 |
| 2 | **KD** | 13.15 | 30.14 | 16.99 | 0.133 | 1.097 |  | 24.24 | 11.09 | 0.270 | 1.206 |  | 20.92 | 7.77 | 1.263 | 2.400 |
|  | **KD** | 13.15 | 30.11 | 16.96 | 0.163 | 1.120 |  | 24.27 | 11.12 | 0.240 | 1.181 |  | 20.86 | 7.71 | 1.323 | 2.502 |
| 3 | **NC** | 13.77 | 31.04 | 17.27 | -0.147 | 0.903 |  | 25.22 | 11.45 | -0.090 | 0.940 |  | 22.91 | 9.14 | -0.107 | 0.929 |
|  | **NC** | 13.75 | 30.29 | 16.54 | 0.343 | 1.268 |  | 25.3 | 11.55 | 0.027 | 1.019 |  | 23.28 | 9.53 | -0.030 | 0.979 |
| 3 | **KD** | 13.36 | 30.17 | 16.81 | 0.313 | 1.242 |  | 24.4 | 11.04 | 0.320 | 1.248 |  | 20.92 | 7.56 | 1.473 | 2.777 |
|  | **KD** | 13.04 | 29.6 | 16.56 | 0.323 | 1.251 |  | 24.22 | 11.18 | 0.397 | 1.317 |  | 20.93 | 7.89 | 1.610 | 3.053 |


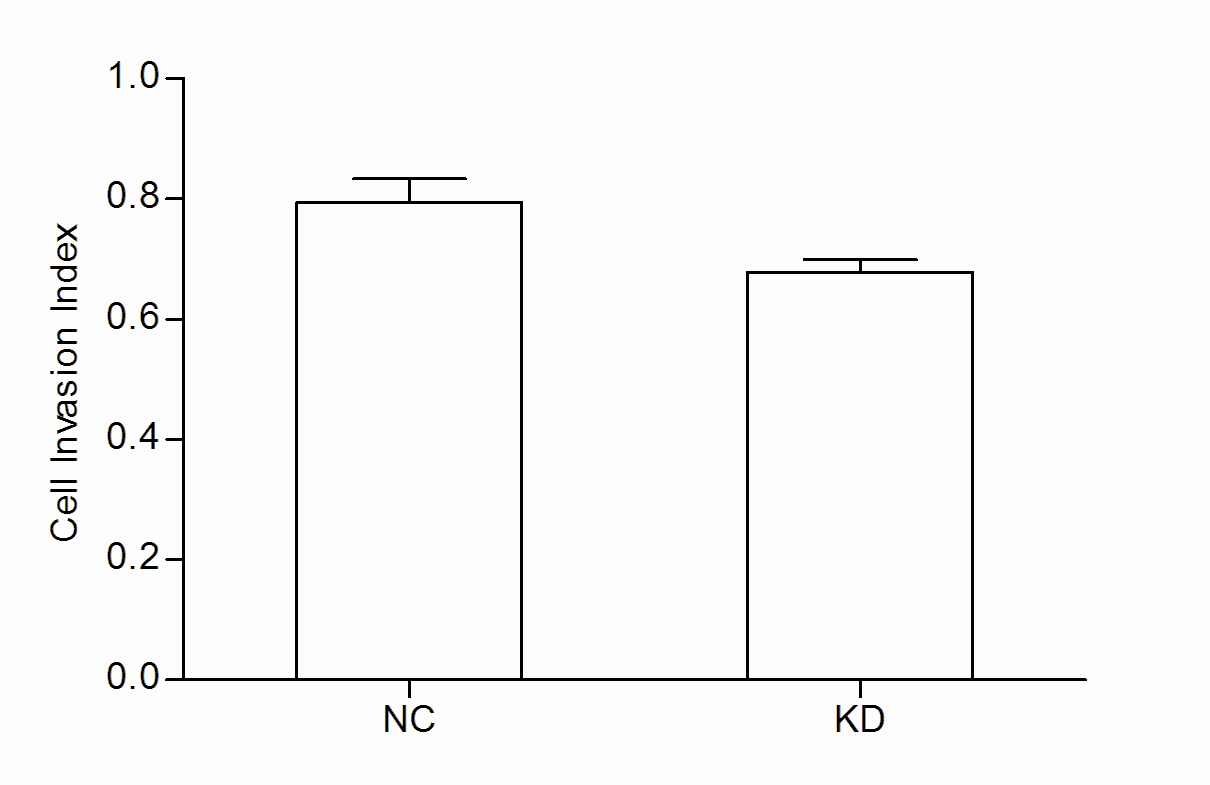


Figure S1: Effect of *H19* knockdown on the invasion of JAR cells. The invasion rate of cells in the *H19* knockdown group and the NC group were 68.85% ± 3.04% and 79.50% ± 5.52%, respectively. *H19* knockdown had no significant impact on the invasion rate of JAR cells.
